# Submesoscale modulation of deep water formation in the Labrador Sea

**DOI:** 10.1038/s41598-020-74345-w

**Published:** 2020-10-15

**Authors:** Filippos Tagklis, A. Bracco, T. Ito, R. M. Castelao

**Affiliations:** 1 Earth and Atmospheric Sciences, Georgia Institute of Technology, Atlanta, GA USA; 2 Department of Marine Sciences, University of Georgia, Athens, GA 30602 USA

**Keywords:** Climate and Earth system modelling, Ocean sciences, Physical oceanography

## Abstract

Submesoscale structures fill the ocean surface, and recent numerical simulations and indirect observations suggest that they may extend to the ocean interior. It remains unclear, however, how far-reaching their impact may be—in both space and time, from weather to climate scales. Here transport pathways and the ultimate fate of the Irminger Current water from the continental slope to Labrador Sea interior are investigated through regional ocean simulations. Submesoscale processes modulate this transport and in turn the stratification of the Labrador Sea interior, by controlling the characteristics of the coherent vortices formed along West Greenland. Submesoscale circulations modify and control the Labrador Sea contribution to the global meridional overturning, with a linear relationship between time-averaged near surface vorticity and/or frontogenetic tendency along the west coast of Greenland, and volume of convected water. This research puts into contest the lesser role of the Labrador Sea in the overall control of the state of the MOC argued through the analysis of recent OSNAP (Overturning in the Subpolar North Atlantic Program) data with respect to estimates from climate models. It also confirms that submesoscale turbulence scales-up to climate relevance, pointing to the urgency of including its advective contribution in Earth systems models.

## Introduction

Oceanic submesoscale currents (SMCs) occur at horizontal scales of the order of 1 km in the form of density fronts, vortices, and filaments in the surface turbulent boundary layer and of topographic wakes throughout the interior^1–4^. Dynamically, SMCs are influenced, but not dominated, by the Earth’s rotation and ocean stratification, which results in order 1 Rossby (*Ro* = *U/fl*) and Froude (*Fr* = *U/Nh*) numbers for these currents (*U* being a characteristic horizontal velocity scale, *l* and *h* horizontal and vertical length scales, *f* the Coriolis frequency, and *N* the Brunt–Vaisala frequency). In the presence of energetic boundary layer currents flowing along steep slopes, topographic wakes may become unstable with consequent generation of coherent vortices that are substantially submesoscale in nature and generated by partially unbalanced turbulence but can have size in the mesoscale (> 10 km) range. These vortices can have a long lifespan (> 1 year), travelling long distances from their point of origin, and their cumulative effect could impact the large scale transport and distribution of heat, nutrients and dissolved gases in the ocean. Few studies have focused on these features so far^1–4^, and their global impact has yet to be shown. Here, we attempt to demonstrate it focusing on the Labrador Sea (LS).

The LS is one of the two major sites of the North Atlantic where deep convection regularly occurs. Intense surface cooling during wintertime weakens the ambient stratification and induces convective mixing in the central LS and over portions of its shelves^5,6^. Convection mixes the surface waters to depths exceeding, in some years, 2000 m^7,8^ and forms a fresh, cold and highly oxygenated water mass, the Labrador Sea Water (LSW). The LSW spreads southward across the northwest Atlantic at mid-depths^9^, is a source to the North Atlantic Deep Water (NADW) and a contributor to the Atlantic portion of the Meridional Overturning Circulation (AMOC).

Despite its oceanographic and climatic importance, and the relatively good observational record^10–12^, both ocean-only and coupled climate models suffer from biases and divergent behaviors in simulating LSW formation and variability at seasonal to decadal timescales^13–15^. A recent analysis^16^ indicates that state-of-the-art climate models run by the three USA national laboratory (NCAR, NASA-GISS and GFDL) overestimate the LSW volume by 60 to 300%.

The surface circulation in the LS is cyclonic and intensified along the boundaries. Near the surface, the West Greenland Current (WGC) and the Labrador Current flow along the continental slopes, northward and southward, respectively. The WGC transports fresh and cold water from the Nordic Seas along the Greenland coast, while the Labrador Current carries cold and fresh water from Baffin Bay towards Nova Scotia. Underneath and offshore of the WGC, the Irminger Current (IC) carries the warmer and saltier Irminger Sea Water (ISW). The IC contributes to restratifying the interior of the basin^17^ and prevents ice formation in the central portion of the basin in winter.

Convective activity to the south of the Greenland tip is controlled by momentum fluxes and wind forcing, while in the central portion of the basin the LSW formation is driven by local surface buoyancy loss and modulated by the atmospheric heat fluxes^18^ and by ocean dynamics^19,20^. Long-lived coherent mesoscale eddies populate the basin and modify the heat and salt budgets of the gyre interior. The largest are the Irminger Rings (IR) with a diameter between 30 and 60 km^19^. They form through localized baroclinic instability^21^ near a constriction of the isobaths along West Greenland, north of Eirik Ridge^22^, are predominantly anticyclonic and transport off-shore the warm and salty water from the IC. As they approach the convective region, they release the heat in their cores and compensate the heat loss from the surface during wintertime convection^19,23,24^.

Several other factors contribute to the LS hydrography and its variability, including freshwater inputs from Arctic and Greenland Ice Sheet (GrIs) melting, continental runoff and precipitation. Over the recent decades, GrIS mass losses accelerated, especially along west Greenland, resulting in increased freshwater inputs into the adjacent seas. Future projections indicate as highly probable a further exponential acceleration^25,26^. Freshwater fluxes contribute to the upper stratification in the LS^27^, also influencing the marine ecosystem primary productivity^28–31^. Recently, global climate models suggested that these freshwater anomalies may reduce dramatically the LS convection, weakening the AMOC^32,33^. Regionally focused experiments at 2.5 km horizontal resolution, on the other hand, have shown that most of the surface meltwater runoff from southwest Greenland is not transported offshore the continental shelf^34^, with strength and direction of the winds in August and September determining the offshore transport of GrIs meltwater^34–36^. Little is known, however, of the contribution that SMCs may have in offshore advection of heat and freshwater anomalies.

In this work we probe how the LSW formation is represented in a regional model, at mesoscale permitting (15 km, LBR15), mesoscale resolving (5 km, LBR5) and submesoscale permitting (1.7 km and 1 km, LBR1.7 and LBR1, respectively) horizontal resolution, focusing on the contributions of submesoscale circulations. The integrations are performed with and without the GrIs meltwater input.

## Methods

We use the Regional Ocean Modeling System (ROMS)^37,38^ in its Coastal and Regional Ocean COmmunity model (CROCO) version^39,40^. ROMS is a free-surface, terrain-following, hydrostatic primitive equation model, configured here loosely following^34^.

Convection in the LS takes place within vertical plumes with radius  of order O(1) km and vertically extent up to 2 km^5^ that can be simulated directly only using non-hydrostatic models^41^. In ROMS the non-local K-Profile Parametrization (KPP) scheme^42^ accounts for the unresolved vertical mixing and realizes the vertical turbulent fluxes as a summation of down-gradient fluxes and non-local contributions. Previous studies have shown that ROMS can properly simulate extent, seasonality and variability of convective episodes whenever the mesoscale circulation of the basin is resolved^18,34,43^.

**Figure 1:**
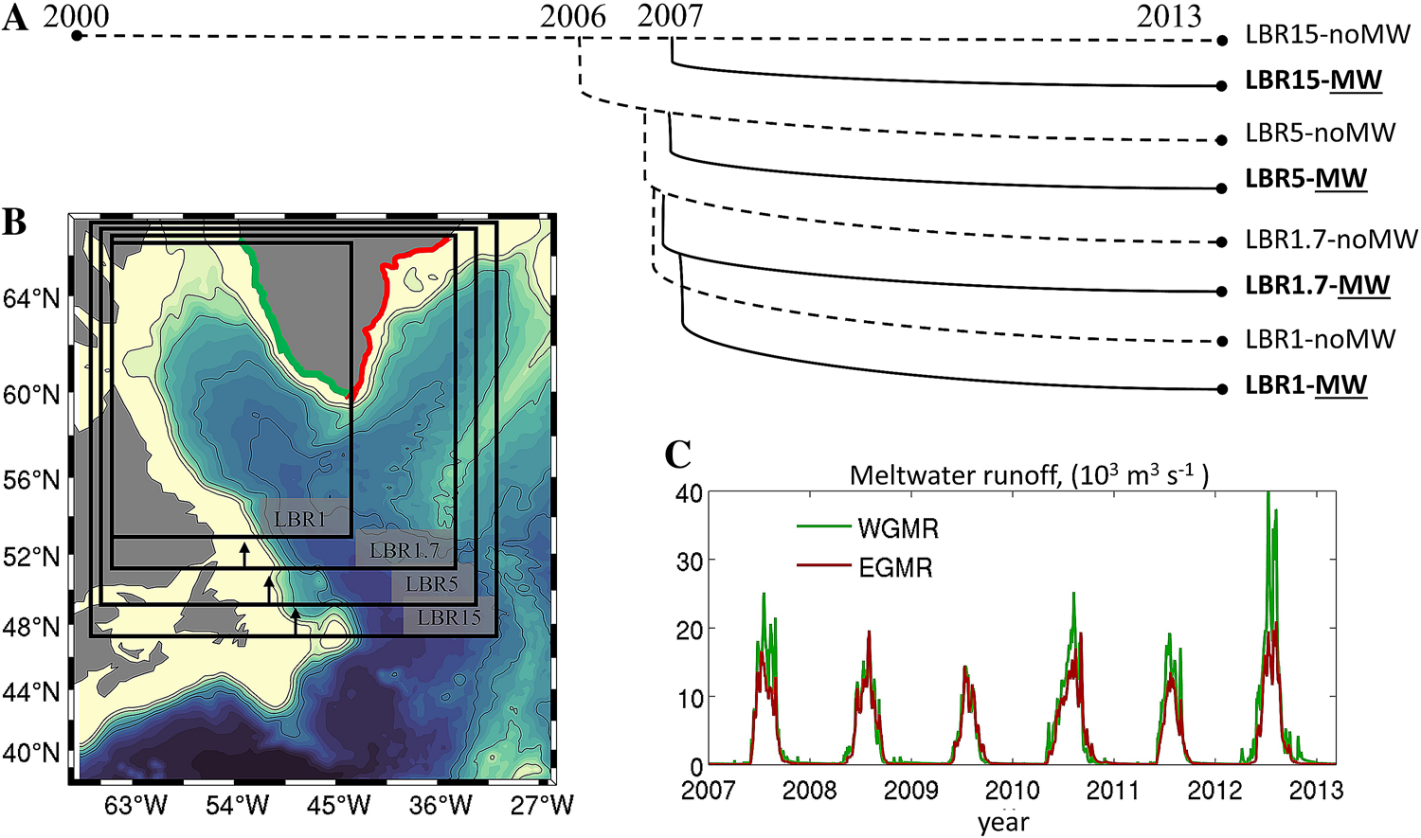
(**A**) Flowchart of experiments. Dashed black lines represent the experiments in the absence of meltwater input from the GrIS (noMW) and solid black lines represent the experiments with meltwater released along the Greenland coast (MW). (**B**) Black squares represent the boundaries of the domains with arrows pointing towards the ensuing nested domain. (**C**) Total GrIs meltwater runoff summing up the sources along the West Greenland coast (WGMR) and East Greenland coast (EGMW).

The horizontal resolution in the study increases from 15 km (LBR15) to 5 km (LBR5), 1.7 km (LBR1.7), and finally 1 km (LBR1), with 30 vertical levels in all cases. We followed the offline-nesting procedure described in^44^ as we moved towards decreasing the grid-point size, starting from the mesoscale permitting case (Fig. 1). The topography is derived from ETOPO2^45^. To avoid potential errors associated with the pressure gradient and the s-coordinate horizontal layers^46^ but capture as much bathymetric detail as possible, the topography is smoothed using a logarithmic interpolation method^47^ to a maximum slope parameter r_max_ = 0.35. The parameter r_max_ is defined as the ratio (r_max_ = Δh/h_mean_) of the maximum difference between adjacent grid cell depths and the mean depth at that point. The bathymetric details are important given the topographic control of the boundary current meandering and eddy formation along the West coast of Greenland, around Cape Desolation^21, 22,48^.

The model domain in the LBR15 case covers approximately 48° N–66.5° N and 34° W–65° W. All boundaries are open and, at the boundaries, the velocity fields, along with temperature and salinity profiles, are nudged to the Simple Ocean Data Assimilation (SODA) reanalysis version 3.4.2^49^. The circulation in the basin is sensitive to the width, strength and variability of incoming currents, and the choice of SODA is supported by a previous comparison with other reanalysis products^43^. In all runs, the model is forced by daily surface winds stresses and heat fluxes from the ERA-interim product^50^ and the surface heat fluxes are corrected towards the NOAA Extended Reconstructed Sea Surface Temperature (ERSST), Version 4^51^, available at (https://psl.noaa.gov/thredds/catalog/Datasets/noaa.ersst.v4/catalog.html). As we increase horizontal resolution, the size of the nested domains decrease and boundary conditions are extracted from the run at the immediately lower resolution, so that LBR1 covers only the Labrador Sea proper (52.5° N–65.5° N and 44° W–64° W) (Fig. 1B). We choose to impose high frequency boundary conditions (3-day averages) to avoid deflections along the southern boundary.

Stratification and horizontal density gradients along the west and east Greenland coasts (west of 47.6° W, east of 44.2° W and south of 66° N) are strongly affected by the seasonal meltwater inflow from the Greenland fjords which is greatest during summer, between July and September. We focus in the period September 2007–August 2013, during which very different momentum and meltwater fluxes conditions were observed. In a first set of runs, the meltwater is included and the input dataset used is that in^34^. The runoff at the 108 major fjords along the whole coast of Greenland is estimated by the Modele Atmospheric Regional (MAR) coupled to the 1-D Surface Vegetation Atmosphere Transfer scheme Soil Ice Snow Vegetation Transfer (SISVAT)^52^. The amount of meltwater introduced from GrIs (Fig. 1C) does not vary among the experiments but the number of source points ‘fjords’ may differ due to grid size limitations. For example, in the lower resolution case the meltwater is introduced at 80 grid points by summing up the discharge amount of two or three fjords within one CROCO grid cell into one source point. A passive tracer is also released at each source point with constant concentration and in each experiment, except for the highest resolution run. At all ‘fjords’, the meltwater and the corresponding passive tracer are injected with a constant vertical profile and among s-layers. In LBR1 the eastern boundary is located at 44° W, thus the meltwater signal originating from the east coast of Greenland is introduced in the LS domain through the eastern open boundary nested onto the LBR1.7 case.

## Results

### Resolution dependency on mean circulation, eddies and vorticity

The mean circulation in the Labrador Sea is well reproduced in all numerical experiments, independently of the horizontal resolution. The maximum transport in the basin is 42 Sv (1 Sv = 10^6^ m^3^ s^−1^) in agreement with previous estimates^53,54^. The mean WGC speed over the Greenland shelf is in the range of 0.2–0.4 m s^−1^, comparable with the observed value of 0.35 m s^−1^ obtained from surface drifters^17^. In the proximity of Hamilton Bank, the simulated Labrador Current speed is also in agreement with observations (~ 0.20 m s^−1^) and is about 0.2–0.4 m s^−1^ onshore (Figure S1). Differences in the mean circulation among experiments can be summarized as follows: the intensity of the WGC, EGC and LC currents increases for increasing resolution; while intensity increases, WGC narrows and in the 1.7 km and 1 km resolution runs the intensification is confined to the core of the current that sits offshore the shelf and away from the fjords. The broader WGC flows westward at 60° N following a steady path along the 3000 m isobath in the mesoscale permitting resolution, while the westward veering begins at lower latitudes, south of Cape Desolation, in the LBR5, LBR1.7 and LBR1 cases.

**Figure 2:**
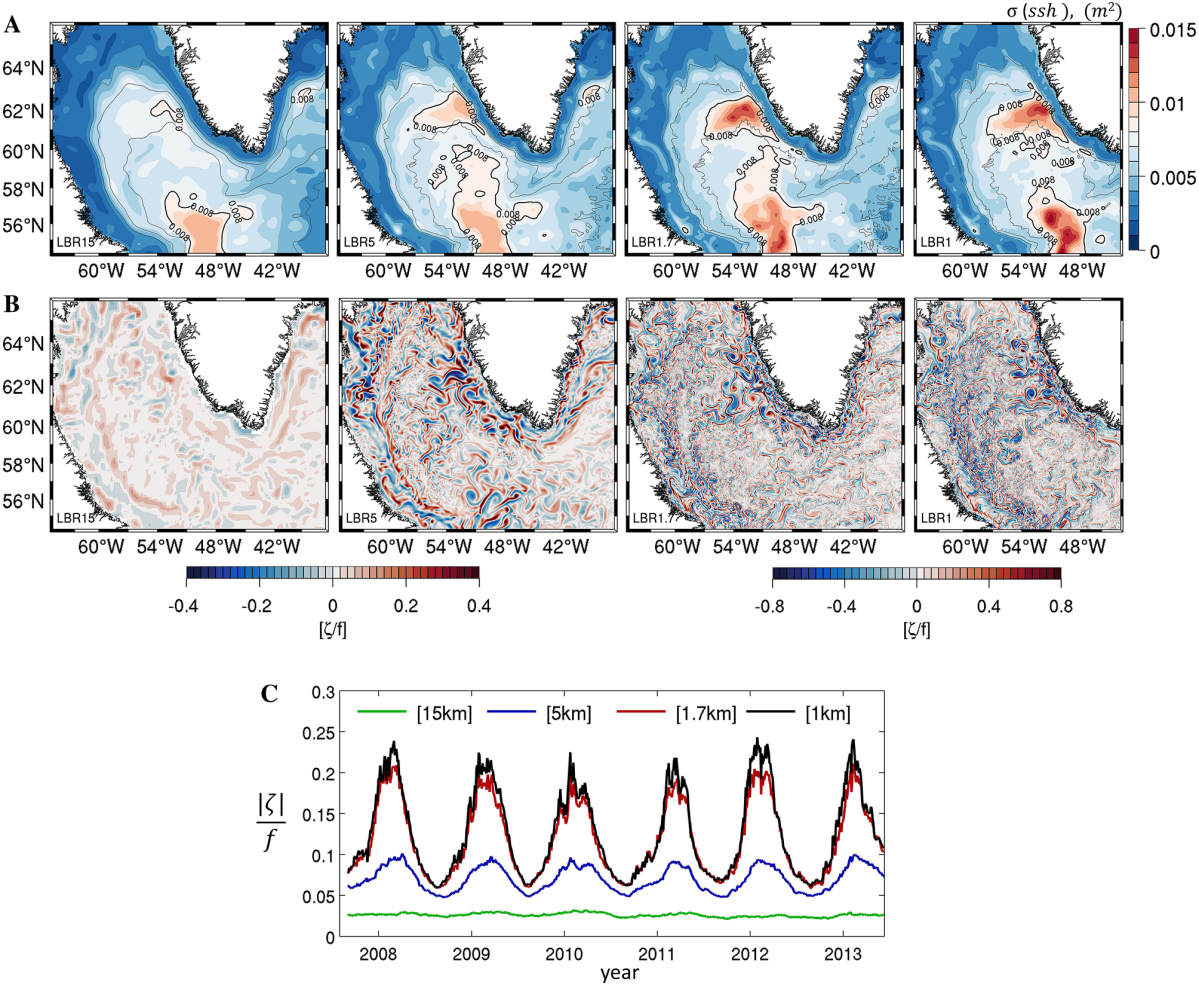
(**A**) Sea surface height variance over the period September 2007–August 2013. Dashed, gray lines represent the 1000 m, 2000 m and 3000 m isobaths. (**B**) Instantaneous snapshots of surface relative vorticity normalized by Coriolis (ζ/f) in February 2009. The color intervals are chosen to highlight the structures but are not representative of the extremum values. Days plotted vary across runs and in the LRB5 and LBR1 cases were selected to have comparable mesoscale circulation offshore West Greenland. (**C**) Fall 2007–summer 2013 time-series of the absolute value of surface ζ/f averaged over LRB1 domain.

In Fig. 2A the variance of the sea surface height averaged over the integration period is presented as a proxy of eddy activity. The maximum variability occurs off the west coast of Greenland, around 61° N and 52° W, and a secondary maximum in the central Labrador Sea is found at about 58° N and 52° W, as in the altimetric data^55^. The strength of the signal in both simulated areas of maximum variability increases with resolution. Surface vorticity differs significantly among simulations, especially in winter, when eddies are more abundant^56^ (Fig. 2B). CROCO captures the formation of the IRs^23,48,57^ only at the three finer resolutions, 5 km, 1.7 km and 1 km; more intense mesoscale eddies, submesoscale coherent vortices (or SCVs) and vorticity filaments^1^ fill the basin in the LBR1.7 and LBR1 simulations, as quantified by the time series of the domain-averaged vorticity (Fig. 2C). SCVs form abundantly where the continental slope meets the shelf which corresponds, on the Greenland side, at the meeting point between the IC and WGC. The time-series of absolute relative vorticity display small and comparable interannual variability in all cases and a seasonal cycle that becomes more pronounced the higher is the resolution, with elevated values from mid-November to May and a less active period during summer and early fall. In the submesoscale permitting cases, the winter amplification follows that found in open ocean studies^58–60^. Noticeably, the increase in vorticity towards the end of summer happens not only faster but also slightly earlier in LBR1.7 and LRB1 compared to the mesoscale resolving case (LBR5).

### Resolution dependency on deep water formation

Next, we explore the horizontal resolution dependency of the representation of deep convection in the LS, spanning different dynamical ranges, from mesoscale to submesoscale permitting. We quantify the impact of horizontal resolution in the representation of LSW formation computing the mixed-layer depth (MLD) over the basin, defined as the depth at which density differs from the surface by 0.008 kg m^−3^, as in^18^. This threshold follows from the observation in^61^ that density differences between the surface and the base of the mixed layer in the LS during convective events can be smaller than 0.01 kg m^−3^, usually adopted in numerical studies^62^. The convective patch is then defined as the area ${{\varvec{A}}}_{{\varvec{M}}{\varvec{L}}{\varvec{D}}>1000{\varvec{m}}}$ where the MLD exceeds 1000 m, and the convective volume V_MLD_ (m^3^) as the volume of water contained within this patch from the surface to the base of the mixed-layer (${{\varvec{V}}}_{{\varvec{M}}{\varvec{L}}{\varvec{D}}}={\int }_{0}^{{{\varvec{M}}{\varvec{L}}{\varvec{D}}}_{{\varvec{B}}{\varvec{a}}{\varvec{s}}{\varvec{e}}}}{{\varvec{A}}}_{{\varvec{M}}{\varvec{L}}{\varvec{D}}>1000{\varvec{m}}}{\varvec{d}}{\varvec{z}}$).

**Figure 3:**
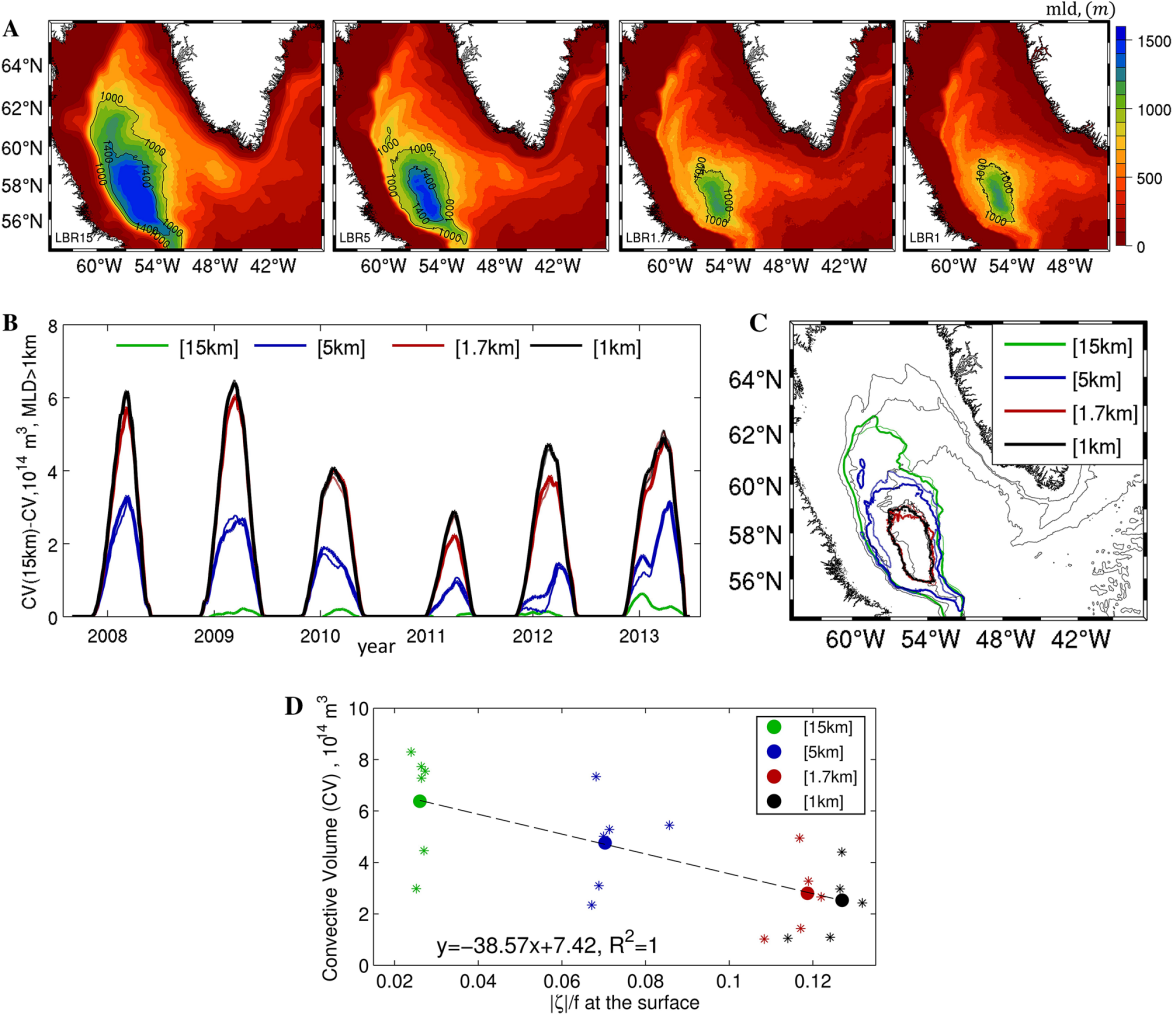
(**A**) 5-year mean (2008–2013) mixed layer depth (in meters) defined using a density criterium 0.008 kg m^−3^ during the convective season (January–May). (**B**) Time series of the difference in convective volume (m^3^) between the LBR15 NoMW case and all other simulations. (**C**) mean (2008–2013, January–May) convective patch, where MLD > 1000 m. Color represents the resolution and thickness indicates the experiment. Thick lines for MW experiments, thin lines for NoMW experiments. (**D**) Mean Jan–May convective volume (CV) and mean absolute value of surface ζ/f averaged over LRB1 domain. Stars represent annual means and circles represent the multi-year (2008–2013) means. The dashed line shows the linear fit based on the multi-year means.

The MLD averaged over the period January–May of each year (2008–2013) is shown in Fig. 3A for all simulations. The modelled convective patches are centered at about [57° W–57° N], consistent with observations^6,63^, but their extent differs significantly among runs. Convective area and convective volume decrease as resolution increases. As previous regional simulations have shown^18^, and in agreement with observational and modelling studies^18,64–67^, temporal characteristics of convective activity as initiation, intensity, seasonality and duration are modulated foremost by the strength of the atmospheric heat fluxes, and secondly by the characteristics of the IC that is advected to the interior of the LS basin. From a numerical point of view, the representation of the boundary current system and its instabilities is important for reproducing correctly such stratification. The IRs, in particular, contribute through eddy-induced lateral fluxes and lateral mixing^19^; however their effectiveness depends greatly on model resolution. In the simulation that barely resolves the Rossby deformation radius of the basin (LBR15), the absence of vigorous mesoscale activity causes a weak stratification of the upper water column in the central portion of the basin, as to be expected, resulting in stronger mixing and a deeper mixed layer patch that extends further north compared to the higher resolution cases (Fig. 3). Resolving the mesoscale circulations (LBR5) induces a nearly 50% reduction of the convective volume produced by LBR15. This is shown in Fig. 3B by the time series of the difference in convective volume between the lowest resolution experiment in the absence of meltwater input (LBR15-NoMW) and all other runs. The meltwater from the GrIS further decreases convection by an additional 2.7% (LBR5-MW). As Fig. 3C suggests, in LBR5, this additional reduction takes place in the northeast corner of the convective patch case (thick versus thin blue lines). The contribution of submesoscale advection amounts to another 30% decrease. Differences between LBR1.7 and LBR1, with and without GrIS input, remain linear. This information is further quantified in Fig. 3D, where the convective volume averaged over the convective season (Jan–May) is plotted against the mean value of near surface vorticity averaged over the LBR1 domain. A linear fit describes well the dependence of the time averaged quantities. It is enlightening to visualize the link between meso- and submeso-scale circulations and MLD also through time-snapshots of MLD, shown for various days in February 2009 in Figure S2.

Previous simulations^34^ at 2.5 km horizontal resolution suggested that salinity anomalies are trapped by the energetic current system along the shoreline and on the shelf, winds are key to exporting the meltwater signal off-shore^34,36^, and the meltwater modulation of deep convection is likely small in current meltwater conditions. This is confirmed by the present experiments, independently of resolution. As the resolution increases towards submesoscale permitting, we observe additional export of meltwater towards the central Labrador Sea. The surface meltwater signal delimits in all cases the edges of the convective area (Figure S3) but has only a small impact on the overall convective activity for a given resolution (Table 1). The impact is inversely proportional to model resolution. Whenever the resolution is low, some GrIS meltwater can be advected into the convective area by the broader and more diffused boundary currents.

**Table 1:** Convective volume as a percentage to the volume produced in the lowest resolution 15 km case in the absence of melt water input from the GrIS (LBR15-NoMW) calculated over the convective period January to May and 2008–2013.

In comparison to LBR 15 km (NoMW)	Convective volume (% LBR 15-NoMW)	
	NoMW	MW
LBR15 (15 km)	100	98.3
LBR5 (5 km)	52.8	50.1
LBR1.7 (1.7 km)	16.4	16.3
LBR1 (1 km)	15.8	15.3

**Figure 4:**
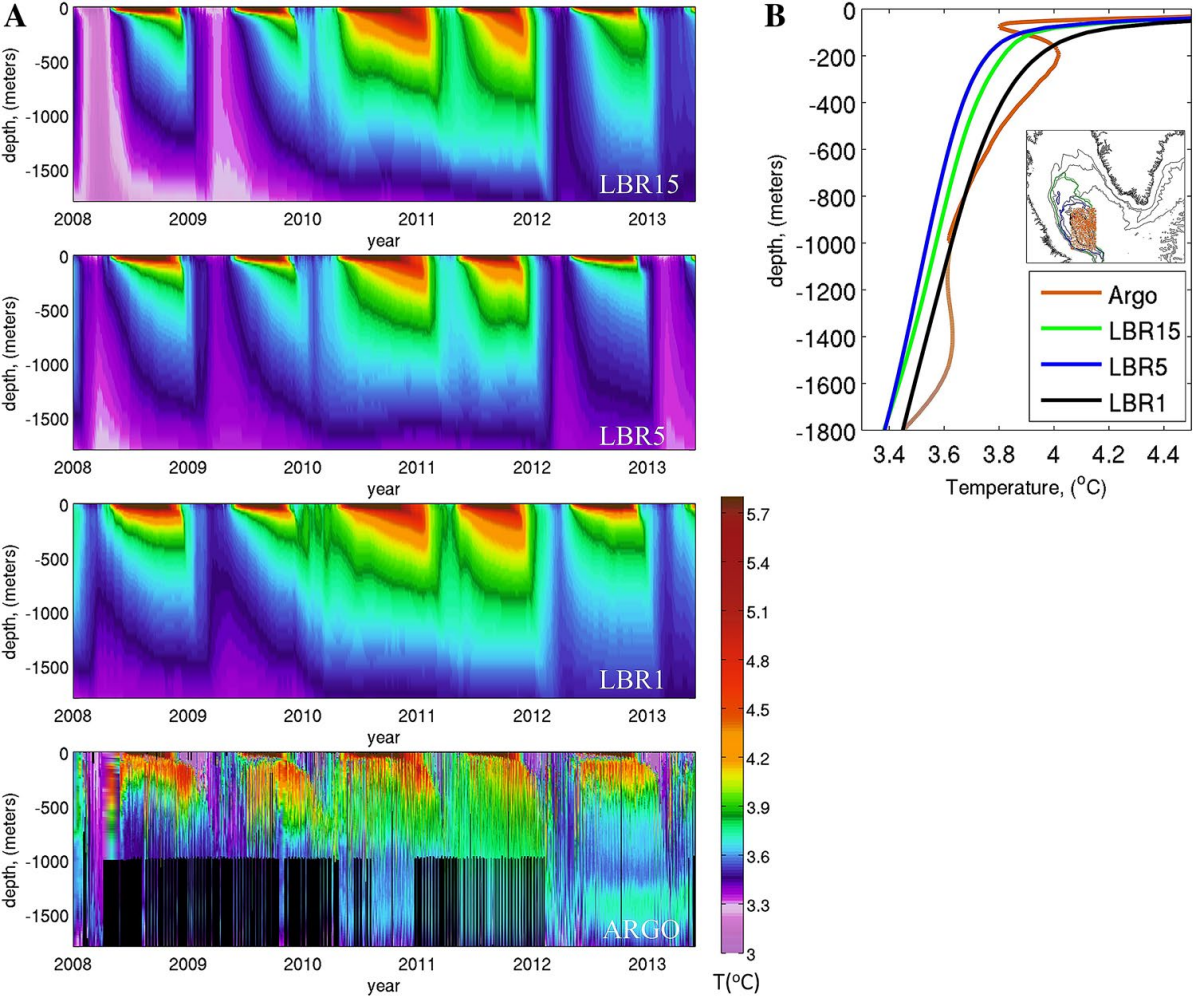
(**A**) Evolution of potential temperature (T, in °C) in the region encompassing the convective area common to all simulations in LBR15, LBR5 and LBR1 and in the ARGO dataset from 2008 to 2013. (**B**) Vertical temperature profile over the same region shown in insert averaged over the entire period. The ARGO portion below 1000 m (orange) is dashed due to the paucity of profiles available. The average number of ARGO profiles in any given month is about 20 and varies between 2 and 70 with a positive trend over time; most profiles do not extend below 1000 m depth.

To verify that the representation of convective activity improves in realism whenever submesoscale processes are included, in Fig. 4A,B we compare the simulated temperature profiles averaged in a region that encompasses the common convective area (MW-experiment) over the integration period, and the same quantity reconstructed from ARGO floats. The average number of ARGO profiles in each month is about 20. The best representation of the mean temperature stratification is provided by LBR1 with LBR1.7 (not shown) being a close second, while a cold bias characterizes the other two resolutions, in line with the greater convective volume. We note the poor representation of the details in the vertical structure found in the observed temperature profiles in the upper 400 m. The limited vertical resolution adopted is likely responsible for it. Indeed, 50 vertical levels would be needed to resolve the first baroclinic mode, with an additional 25 levels per subsequent mode^68^ .

**Figure 5:**
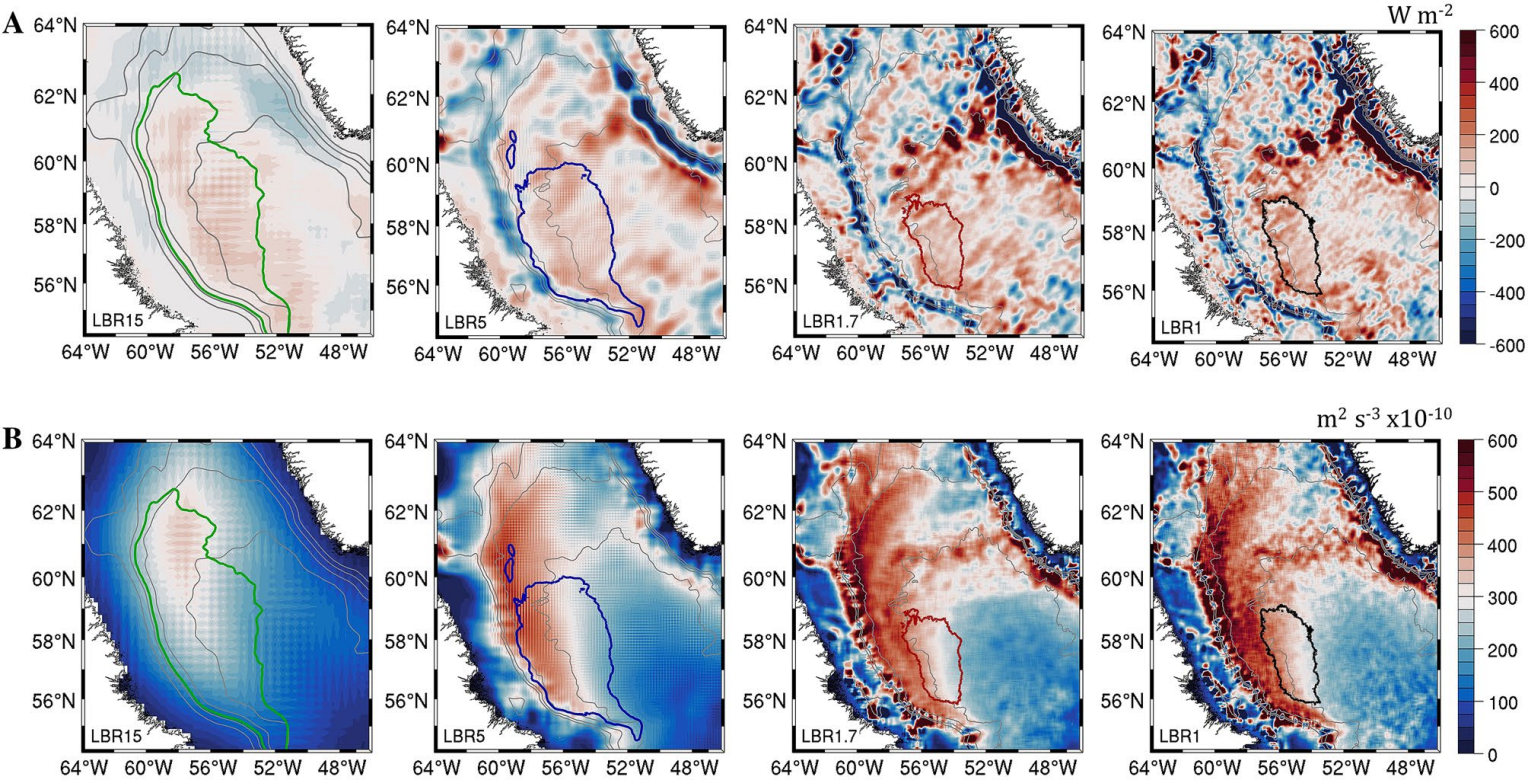
(**A**) Depth integrated eddy heat advection (W m^−2^). Positive/negative values translate to heat convergence/divergence and temperature—and therefore stratification—increase/decrease. (**B**) Depth integrated vertical eddy flux of buoyancy, $\frac{1}{{\text{H}}}\int\limits_0^H {\overline {{{\text{w}}^\prime }{{\text{b}}^\prime }} {\text{dz}}}$ over the top H = 400 m. Thick colored contours represent the mean convective region (Fig. 3C) and thin grey lines represent the 500-m, 1- , 2- and 3-km isobaths.

The very small differences between the integrations with and without meltwater suggest that the salinity anomalies linked to the GrIs do not contribute significantly (yet) to the stratification of the LS interior. Differences in overall heat transport must therefore drive the resolution dependence by controlling the stratification in the central portion of the basin; alternatively or additionally, submesoscale eddies formed locally in the central LS may restratify the water column in winter and impede convection. To verify both possibilities, in Fig. 5 we evaluate the eddy contribution to the heat transport and the local available potential energy release in winter among the MW experiments. In Fig. 5A the depth-integrated eddy advection (E) of heat is presented for the four different resolutions. Eddy advective fluxes are defined as ${\varvec{E}}=-\nabla (\overline{{\varvec{u}}{^{\prime}}{\varvec{C}}{^{\prime}})}-{\partial }_{{\varvec{z}}}\overline{{{\varvec{w}}}{^{\prime}}{{\varvec{C}}}{^{\prime}}}$ and the depth-integration extends to the whole water column. Prime values indicate the deviation from the average over the whole period (2007–2013), and the overbar indicates again their time average. ∇ is the 2 dimensional gradient operator; **u**(u,v) and w are the horizontal and vertical components of the velocity field, respectively; $C={\rho }_{o}{c}_{p}\theta$ is the heat content, and ρ_ο_, c_p_ and θ are the reference density, specific heat, and potential temperature of water. Mean advective fluxes, defined as $M=-\stackrel{-}{{\varvec{u}}}\nabla \overline{C}-\overline{w}{\partial }_{z}\overline{C}$ can be found in Figure S5. The spatial structure of both net mean and eddy contribution is far more complex in the three finer resolutions (LBR5, LBR1.7 and LBR1) than in LBR15 where a local maximum cannot be distinguished, but the integral of the net mean contribution where convection can take place—the LBR15 convective patch—does not vary significantly across resolutions. For the eddy component (Fig. 5A), on the other hand, very high values of E are located off the Greenland coast, in the region of eddy formation, in the mesoscale resolving and submesoscale permitting simulations (LBR5, LBR1.7 and LBR1), consistent with^57, 69^. Furthermore, in LBR1.7 and LBR1 the convergence of heat by eddy advection is large also in the central Labrador Sea following the IR path, with the maximum values reaching E > 500 W m^−2^ to the north-northeast of the LBR1.7 and LBR1 convective patches. This heat convergence therefore delimits the convective patch in the submesoscale permitting runs by controlling the stratification in the off-shore portion of the basin. The time-mean eddy contribution to heat advection over the LBR5 convective area is 70 W m^−2^ and over the same area increases to 83 and finally 90 W m^−2^ in LBR1.7 and LBR1, respectively.

Submesoscale circulations, and specifically mixed-layer eddies, can also contribute to restratifying locally the upper portion of the water column^70^, and this contribution can be parameterized in climate models^71^. This local restratification mechanism can be evaluated through the available potential energy (APE) release in the convective season, from December to April (Fig. 5B). Such release is estimated by the vertical eddy flux of buoyancy, $\overline{w{^{\prime}}b{^{\prime}}}$*,* where *b* is buoyancy, here integrated over the upper 400 m of the water column, given the vertical extent of the submesoscale circulations and the core of the IR (see Fig. 7 below). The APE release is small in LBR15 everywhere but in the convective area, as to be expected, and increases by increasing resolution. Such increase is strong along the western portion of the basin between the 2000 and 3000 m isobaths in all other runs, and also along the IR path and the Greenland slope in LBR1.7 and LBR1. The APE release inside each respective convective area does not vary significantly or linearly between mesoscale resolving and submesoscale permitting runs (149, 146 and 158 × 10^–10^ m^2^ s^−3^ from LRB5 to LBR1), but increases steadily by 20 and 30 × 10^–10^ m^2^ s^−3^ in the region comprised between the western boundary of the LBR1.7 and LBR1 patches and the 2000 m isobath to the west of it.

Overall, the latitudinal shrinking of the convective patch for increasing model resolution is dominated by the eddy-driven heat convergence, while the longitudinal shrinking is modulated by both mechanisms, with the local restratification induced by the numerous submesoscale eddies (see Fig. 2) playing an important role in the area comprised between the 2000 and 3000 m isobaths.

**Figure 6:**
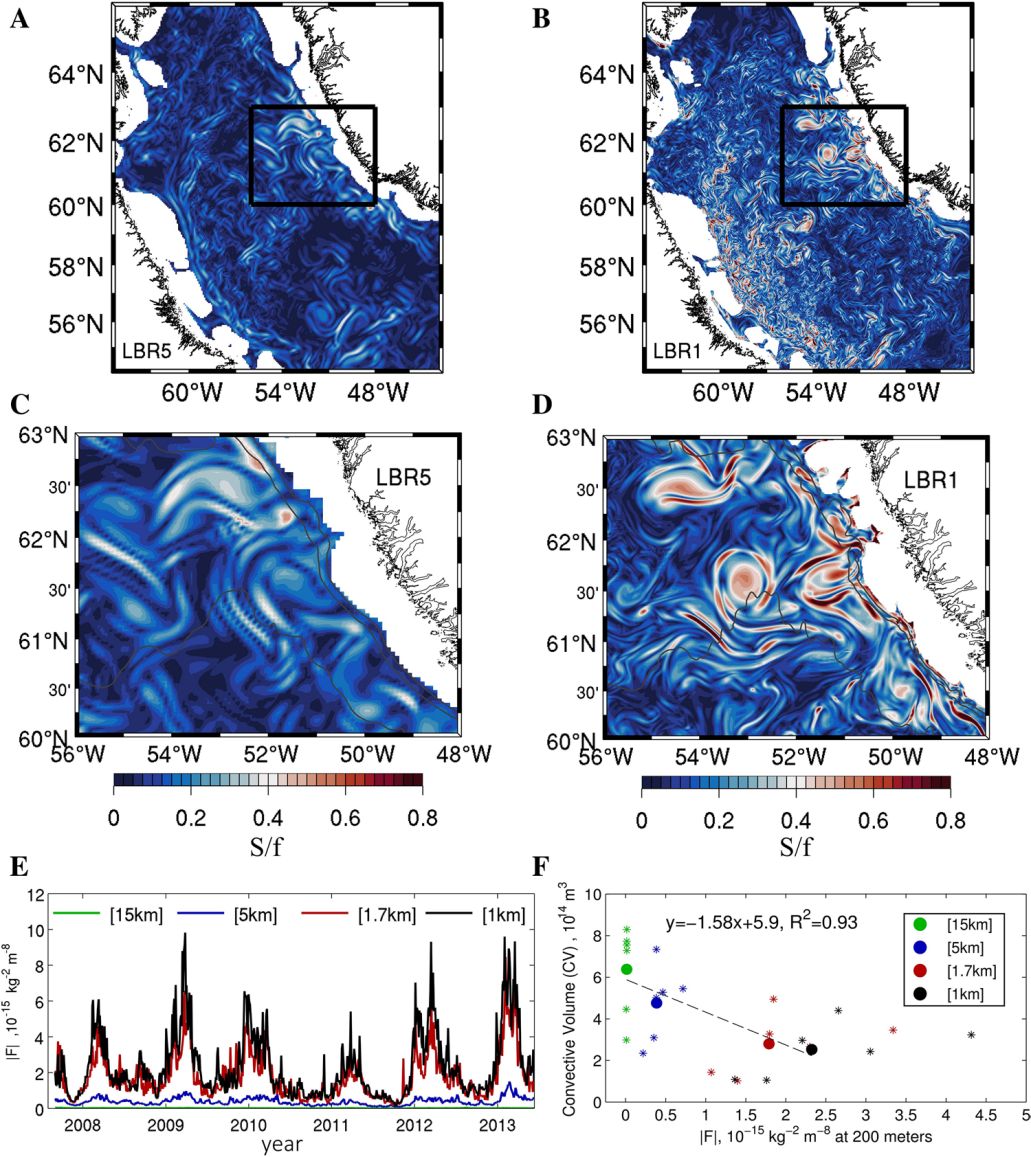
(**A**,**B**) Instantaneous snapshots of strain rate normalized by Coriolis (S/f) in the LBR5 and LBR1 simulations, respectively (times as in Fig. 1). High values, accompanied by a high frontogenetic tendency (Fig. S2), are indicative of submesoscale dynamics. (**C**,**D**) Zoom of (**A**,**B**) over the constriction of the isobaths along West Greenland, north of Eirik Ridge, where IRs are formed. (**E**) Time series of frontogenetic tendency |F| averaged over the LBR1 domain for the MW experiments. (**F**) Mean Jan–May convective volume (CV) plotted against mean absolute value of F at 200 m depth averaged over the domain of (**C**,**D**). Stars represent annual mean values and dots represent the multi-year (2008–2013) mean. The dashed line shows the linear fit for the multi-year mean values.

**Figure 7:**
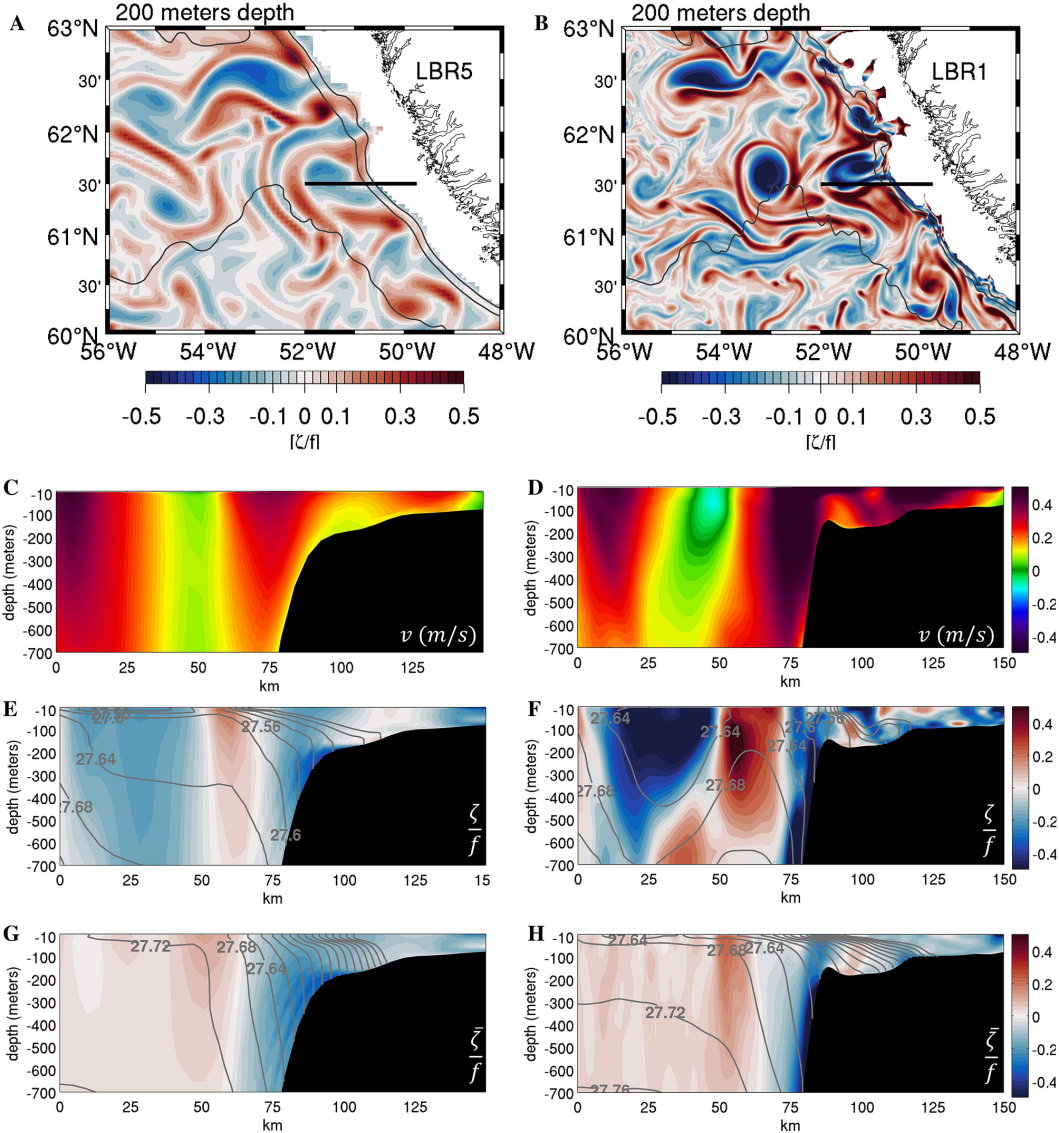
Left column LBR5 and right column LBR1 simulations. (**A**,**B**) Relative vorticity normalized by Coriolis (ζ/f) at 200 m depth in wintertime zoomed over the constriction of the isobaths along West Greenland where IRs are formed. (**C**,**D**) Snapshot of meridional velocity in m s^−1^ along a transect at 61.5° N (black line in **A**,**B** panels) (times as in Fig. 1). (**E**,**F**) Snapshots of ζ/f with density isolines superposed. (**G**,**H**) ζ/f across the transect time-averaged over the 2009 convective season with corresponding density isolines superimposed.

While the meltwater signal is confined near the surface and does not influence convection strongly, the advection pathways of the meltwater from Greenland into the interior provide another indication of the processes at play. In the submesoscale permitting cases, advection from the boundary currents is accomplished by the long-living population of anticyclonic vortices and cyclonic vorticity filaments characterized by *Ro* of order 1. The submesoscale process behind the subsurface intensification of these circulations in LBR1 or LBR1.7 compared to the lower resolution cases is, predominantly, strain-induced frontogenesis^72^ along the continental slope. This is supported by the snapshots of the strain field at 200 m of depth, defined as $S={\left[{\left(\frac{\partial u}{\partial x}-\frac{\partial v}{\partial y}\right)}^{2}+{\left(\frac{\partial v}{\partial x}-\frac{\partial u}{\partial y}\right)}^{2}\right]}^{1/2}$ and by the time-series of frontogenetic tendency, defined as $F=\frac{D({\nabla }_{h}\rho )}{Dt} =Q{\nabla }_{h}\rho$ with $Q=\left({Q}_{1,}{Q}_{2}\right)=-\left(\frac{\partial u}{\partial x}\frac{\partial \rho }{\partial x}+\frac{\partial v}{\partial x}\frac{\partial \rho }{\partial y},\frac{\partial u}{\partial y}\frac{\partial \rho }{\partial x}+\frac{\partial v}{\partial y}\frac{\partial \rho }{\partial y}\right)$^73,74^ where *u* and *v* are the horizontal velocity components and ρ is density, shown in Fig. 6. The generation of fronts, or frontogenesis, whenever F > 0, is indicative of the break-down of geostrophic balance and therefore submesoscale dynamics. The IRs in both LBR5 and LBR1.7/LBR1 simulations form near the separation point between the shallow coastal shelf and the deep continental slope along the west Greenland coast by baroclinic instability. They extend vertically to about 400–500 m, encompassing the IC core located between 100 and 300 m depth and their generation and overall impacts are independent of the meltwater input. They form in correspondence of the steepening of the bathymetry and result from baroclinic instability of the horizontal shear layers induced by the warm IC that meets the steep continental slope^24^. Resolution influences their characteristics in two ways. First, the steepness and veering of the actual bathymetry are better resolved in the LBR1 (and LBR1.7) than in LBR5 (or LBR15), contributing to the confinement, intensification and increased variability of the IC (Fig. 7C,D). Second, the horizontal shear layer coinciding with a non-zero vertical component of the vorticity tensor that extends into the interior, because the interior mean flow has to be identically zero at the sloping bottom, is better resolved and intensified through strain-induced frontogenesis (Fig. 6C,D, Figure S4 and Video VS1) in the submesoscale permitting cases. In sum, in LBR1 and LBR1.7 this horizontal shear layer is more intense and has an elevated positive frontogenic tendency. There is indeed a nearly perfect linear relationship also between the frontogenetic tendency along the coast of Greenland at 200 m depth and the convective volume, similarly to what seen for surface vorticity (Fig. 6F). The end result is the formation of eddies of size comparable to the mesoscale case, being the size controlled by the bathymetry^21,24^, but of submesoscale strength (*Ro* ~ 1) being originated from the submesoscale fronts (Fig. 7E–H). The eddies are then surface-intensified by the strong and variable winds. The IRs in the LBR1.7 and LBR1 simulation are not only stronger, but they are also longer living that the LBR5 counterpart, are surrounded by submesoscale filaments, and, together with the filaments, contribute more effectively to the advection of warm water into the center of the LS. They form abundantly especially in late fall and winter, when the winds and the boundary current are stronger and more variable^56^, and they stratify the portion of the basin they travel to, effectively delimiting the convective patch and its volume. In this regard, Figure S3, showing the distribution of passive dye released at the fjords at the various resolution is illuminating. In doing so, they also transport freshwater originated from the GrIS melting, but the freshwater anomaly remains within the stratified region, where convection does not occur anyway, because of the IC heat contribution and the submesoscale eddy-induced restratification.

## Discussion

Our findings support the existence of a direct link between submesoscale instabilities and the Labrador Sea contribution to the global meridional overturning circulation. Model results reveal surprisingly large differences in the representation of Labrador Sea Water formation for varying resolution, with a 50% reduction in the modeled volume of convected waters (calculated over the area where convection reaches depths greater than 1000 m) when mesoscale advection is included, and over 80% reduction if submesoscale processes are accounted for. Current state-of-the-art climate models do not fully resolve the mesoscale dynamics at high latitudes, and indeed tend to greatly overestimate the formation of Labrador Sea water^16^. Interestingly, the model predicts a linear relationship between the amount of LSW formed, and the mean near-surface vorticity and/or the frontogenetic tendency along the west coast of Greenland, opening the possibility to a physically based parameterization of this contribution.

In the submesoscale permitting simulations, mesoscale anticyclonic eddies with submesoscale characteristics (in primis a Rossby number of order 1) and submesoscale cyclonic vorticity filaments form in correspondence of the intense horizontal shear layers induced by the Irminger Current interacting with the continental slope. These long-lived eddies (longer-lived and more intense than their counterpart in the mesoscale-resolving simulation) carry heat from the Irminger Current to the interior, effectively delimiting where deep convection takes place. They form independently of the near surface stratification along the Greenland coast, their generation being controlled by baroclinic instability associated with the bathymetry and not by the presence/absence of freshwater inputs from the Arctic and Greenland Ice Sheet. At the same time, the large number of submesoscale eddies formed locally in the region bounded by the 2000 m and 3000 m isobaths in the western portion of the basin controls locally the stratification, limiting the lateral extension of the convective patch towards the Labrador shelf.

This work provides physical context to the recent attribution of the differences in sea-level rise between the penultimate and last deglaciation to differences in subsurface warming in the North Atlantic subpolar gyre^75^. It also helps understanding the lesser role of the Labrador Sea in the overall control of the state of the MOC argued through the analysis of recent OSNAP (Overturning in the Subpolar North Atlantic Program) data with respect to estimates from climate models^19,76^, while indicating that the representation of the interannual variability of the LSW formation can be captured independently of resolution.

Ultimately, these findings call for observational efforts aimed at carefully monitoring heat content and trends of the Irminger Current and point to the need for better investigating the rich complexity of interactions and feedbacks between processes localized at the ocean boundaries (in this case along the continental slope of West Greenland) and the global ocean, as they can affect the climate trajectory of our planet. They also call for parameterizations in Earth system models that account for the advective role of coherent vortices generated by submesoscale instabilities in regions where submesoscale processes cascade to the larger (from basin to global) scales. For the LSW this can be easily achieved by accounting for the linear relationship between convective volume and surface mean vorticity or frontogenetic tendency. Predicting the future evolution of the meridional overturning circulation and its persistence as global climate change progresses will hinge upon our ability to model correctly the response of a multiscale environment.

## Supplementary information

Supplementary Information.

Supplementary Video VS1.

## Data Availability

The numerical data generated and analysed that support our findings can be accessed on-line at https://www.dropbox.com/sh/qz7zw2ia57niot6/AACyxyCr19KZ1ZOddvLsRVbTa?dl=0. The Argo profiles used are available by the EN4 data set^77^ (https://www.metoffice.gov.uk/hadobs/en4/), downloaded on the 2 February, 2020.

## Code availability

All simulations have been performed using CROCO, available at https://www.croco-ocean.org/.

## References

[1] McWilliams JC (2016). Submesoscale currents in the ocean. Proc. R. Soc. A Math. Phys. Eng. Sci..

[2] Srinivasan K, McWilliams JC, Molemaker MJ, Barkan R (2019). Submesoscale vortical wakes in the lee of topography. J. Phys. Oceanogr..

[3] Molemaker MJ, McWilliams JC, Dewar WK (2015). Submesoscale instability and generation of mesoscale anticyclones near a separation of the california undercurrent. J. Phys. Oceanogr..

[4] Bracco A, Choi J, Joshi K, Luo H, McWilliams JC (2016). Submesoscale currents in the northern Gulf of Mexico: Deep phenomena and dispersion over the continental slope. Ocean Model..

[5] Marshall J, Schott F (1999). Open-ocean convection: Observations, theory, and models. Rev. Geophys..

[6] Pickart RS, Torres DJ, Clarke RA (2002). Hydrography of the Labrador sea during active convection. J. Phys. Oceanogr..

[7] Lazier J (1988). Temperature and salinity changes in the deep Labrador Sea, 1962–1986. Deep Sea Res. Part A Oceanogr. Res. Pap..

[8] Yashayaev I (2007). Hydrographic changes in the Labrador Sea, 1960–2005. Prog. Oceanogr..

[9] McCartney MS, Talley LD (1982). THE sub-polar mode water of the north-Atlantic ocean. J. Phys. Oceanogr..

[10] Sathiyamoorthy S, Moore GWK (2002). Buoyancy flux at ocean weather station Bravo. J. Phys. Oceanogr..

[11] Lazier J (1980). Oceanographic conditions at Ocean Weather Ship Bravo, 1964–1974. Atmos. Ocean.

[12] Marshall J (1998). The Labrador Sea deep convection experiment. Bull. Am. Meteorol. Soc..

[13] Canuto VM, Howard A, Hogan P, Cheng Y, Dubovikov MS, Montenegro LM (2004). Modeling ocean deep convection. Ocean Model..

[14] Danabasoglu G (2014). North Atlantic simulations in coordinated ocean-ice reference experiments phase II (CORE-II). Part I: Mean states.. Ocean Model..

[15] Danabasoglu G (2016). North Atlantic simulations in coordinated ocean-ice reference experiments phase II (CORE-II). Part II: Inter-annual to decadal variability. Ocean Model..

[16] Li F (2019). Local and downstream relationships between Labrador sea water volume and north Atlantic meridional overturning circulation variability. J. Clim..

[17] Cuny J, Rhines PB, Niiler PP, Bacon S (2002). Labrador sea boundary currents and the fate of the Irminger sea water. J. Phys. Oceanogr..

[18] Luo H, Bracco A, Zhang F (2014). The seasonality of convective events in the Labrador sea. J. Clim..

[19] Lilly JM (2003). Observations of the Labrador Sea eddy field. Prog. Oceanogr..

[20] Straneo F (2006). Heat and freshwater transport through the central Labrador Sea. J. Phys. Oceanogr..

[21] Bracco A, Pedlosky J (2003). Vortex generation by topography in locally unstable baroclinic flows. J. Phys. Oceanogr..

[22] Katsman CA, Spall MA, Pickart RS (2004). Boundary current eddies and their role in the restratification of the Labrador Sea. J. Phys. Oceanogr..

[23] Hatun H, Eriksen CC, Rhines PB (2007). Buoyant eddies entering the Labrador Sea observed with gliders and altimetry. J. Phys. Oceanogr..

[24] Bracco A, Pedlosky J, Pickart RS (2008). Eddy formation near the West coast of Greenland. J. Phys. Oceanogr..

[25] Hanna E (2008). Increased runoff from melt from the greenland ice sheet: A response to global warming. J. Clim..

[26] Fettweis X (2013). Estimating the Greenland ice sheet surface mass balance contribution to future sea level rise using the regional atmospheric climate model MAR. Cryosphere.

[27] Boning CW, Behrens E, Biastoch A, Getzlaff K, Bamber JL (2016). Emerging impact of Greenland meltwater on deepwater formation in the North Atlantic Ocean. Nat. Geosci..

[28] Arrigo KR (2017). Melting glaciers stimulate large summer phytoplankton blooms in southwest Greenland waters. Geophys. Res. Lett..

[29] Hopwood MJ (2018). Non-linear response of summertime marine productivity to increased meltwater discharge around Greenland. Nat. Commun..

[30] Oliver H (2018). Exploring the potential impact of greenland meltwater on stratification, photosynthetically active radiation, and primary production in the Labrador Sea. J. Geophys. Res. Oceans.

[31] Cape MR, Straneo F, Beaird N, Bundy RM, Charette MA (2019). Nutrient release to oceans from buoyancy-driven upwelling at Greenland tidewater glaciers. Nat. Geosci..

[32] Caesar L, Rahmstorf S, Robinson A, Feulner G, Saba V (2018). Observed fingerprint of a weakening Atlantic Ocean overturning circulation. Nature.

[33] Rahmstorf S (2015). Exceptional twentieth-century slowdown in Atlantic Ocean overturning circulation. Nat. Clim. Change.

[34] Luo H (2016). Oceanic transport of surface meltwater from the southern Greenland ice sheet. Nat. Geosci..

[35] Myers PG (2005). Impact of freshwater from the Canadian Arctic Archipelago on Labrador Sea Water formation. Geophys. Res. Lett..

[36] Schulze Chretien LM, Frajka-Williams E (2018). Wind-driven transport of fresh shelf water into the upper 30 m of the Labrador Sea. Ocean Sci..

[37] Shchepetkin AF, McWilliams JC (2003). A method for computing horizontal pressure-gradient force in an oceanic model with a nonaligned vertical coordinate. J. Geophys. Res..

[38] Shchepetkin AF, McWilliams JC (2005). The regional oceanic modeling system (ROMS): A split-explicit, free-surface, topography-following-coordinate oceanic model. Ocean Model..

[39] Debreu L, Vouland C, Blayo E (2008). AGRIF: Adaptive grid refinement in Fortran. Comput. Geosci..

[40] Debreu L, Marchesiello P, Penven P, Cambon G (2012). Two-way nesting in split-explicit ocean models: Algorithms, implementation and validation. Ocean Model..

[41] Sun D, Ito T, Bracco A (2017). Oceanic uptake of oxygen during deep convection events through diffusive and bubble-mediated gas exchange. Glob. Biogeochem. Cycles.

[42] Large WG, McWilliams JC, Doney SC (1994). Oceanic vertical mixing: A review and a model with a nonlocal boundary layer parameterization. Rev. Geophys..

[43] Luo H, Bracco A, Yashayaev I, Di Lorenzo E (2012). The interannual variability of potential temperature in the central Labrador Sea. J. Geophys. Res..

[44] Mason E, Molemaker J, Shchepetkin AF, Colas F, McWilliams JC, Sangrà P (2010). Procedures for offline grid nesting in regional ocean models. Ocean Model..

[45] Smith WHF, Sandwell DT (1997). Global sea floor topography from satellite altimetry and ship depth soundings. Science.

[46] Beckmann A, Haidvogel DB (1993). Numerical simulation of flow around a tall isolated seamount. Part I: Problem formulation and model accuracy. J. Phys. Oceanogr..

[47] Penven P, Marchesiello P, Debreu L, Lefèvre J (2008). Software tools for pre- and post-processing of oceanic regional simulations. Environ. Model. Softw..

[48] Eden C, Böning C (2002). Sources of Eddy kinetic energy in the Labrador Sea. J. Phys. Oceanogr..

[49] Carton JA, Chepurin GA, Chen L (2018). SODA3: A new ocean climate reanalysis. J. Clim..

[50] Dee DP (2011). The ERA-Interim reanalysis: Configuration and performance of the data assimilation system. Quart. J. R. Meteorol. Soc..

[51] Huang B (2015). Extended reconstructed sea surface temperature version 4 (ERSSTv.4). Part I: Upgrades and intercomparisons. J. Clim..

[52] Gallée H, Schayes G (1994). Development of a three-dimensional meso-γ primitive equation model: Katabatic winds simulation in the area of Terra Nova Bay, Antarctica. Mon. Weather Rev..

[53] Reynaud TH, Weaver AJ, Greatbatch RJ (1995). SUMMER mean circulation of the northwestern Atlantic-ocean. J. Geophys. Res.-Oceans.

[54] Fischer J, Schott FA, Dengler M (2004). Boundary circulation at the exit of the Labrador Sea. J. Phys. Oceanogr..

[55] Prater MD (2002). Eddies in the Labrador Sea as observed by profiling RAFOS floats and remote sensing. J. Phys. Oceanogr..

[56] Luo H, Bracco A, Di Lorenzo E (2011). The interannual variability of the surface eddy kinetic energy in the Labrador Sea. Prog. Oceanogr..

[57] Chanut J (2008). Mesoscale eddies in the Labrador Sea and their contribution to convection and restratification. J. Phys. Oceanogr..

[58] Mensa JA, Garraffo Z, Griffa A, Özgökmen TM, Haza A, Veneziani M (2013). Seasonality of the submesoscale dynamics in the Gulf Stream region. Ocean Dyn..

[59] Callies J, Ferrari R, Klymak JM, Gula J (2015). Seasonality in submesoscale turbulence.. Nat. Commun..

[60] Brannigan L, Marshall DP, Naveira-Garabato A, George Nurser AJ (2015). The seasonal cycle of submesoscale flows. Ocean Model..

[61] Frajka-Williams E, Rhines PB, Eriksen CC (2014). Horizontal stratification during deep convection in the Labrador Sea. J. Phys. Oceanogr..

[62] Lazier J, Hendry R, Clarke A, Yashayaev I, Rhines P (2002). Convection and restratification in the Labrador Sea, 1990–2000. Deep-Sea Res. Part I-Oceanogr. Res. Pap..

[63] Vage K (2009). Surprising return of deep convection to the subpolar North Atlantic Ocean in winter 2007–2008. Nat. Geosci..

[64] Delworth TL, Greatbatch RJ (2000). Multidecadal thermohaline circulation variability driven by atmospheric surface flux forcing. J. Clim..

[65] Eden C, Willebrand J (2001). Mechanism of interannual to decadal variability of the North Atlantic circulation. J. Clim..

[66] Bentsen M, Drange H, Furevik T, Zhou T (2004). Simulated variability of the Atlantic meridional overturning circulation. Clim. Dyn..

[67] Holte J, Talley LD, Gilson J, Roemmich D (2017). An Argo mixed layer climatology and database. Geophys. Res. Lett..

[68] Stewart KD (2017). Vertical resolution of baroclinic modes in global ocean models. Ocean Model..

[69] Saenko OA, Dupont F, Yang D, Myers PG, Yashayaev I, Smith GC (2014). Role of resolved and parameterized eddies in the Labrador Sea balance of heat and buoyancy. J. Phys. Oceanogr..

[70] Boccaletti G, Ferrari R, Fox-Kemper B (2007). Mixed layer instabilities and restratification. J. Phys. Oceanogr..

[71] Fox-Kemper B, Ferrari R, Hallberg R (2008). Parameterization of mixed layer eddies. Part I: Theory and diagnosis. J. Phys. Oceanogr..

[72] McWilliams JC, Gula J, Molemaker MJ, Renault L, Shchepetkin AF (2015). Filament frontogenesis by boundary layer turbulence. J. Phys. Oceanogr..

[73] Hoskins BJ (1982). The mathematical theory of frontogenesis. Annu. Rev. Fluid Mech..

[74] Capet X, McWilliams JC, Mokemaker MJ, Shchepetkin AF (2008). Mesoscale to submesoscale transition in the California current system. Part I: Flow structure, eddy flux, and observational tests. J. Phys. Oceanogr..

[75] Clark PU (2020). Oceanic forcing of penultimate deglacial and last interglacial sea-level rise. Nature.

[76] Lozier MS (2019). A sea change in our view of overturning in the subpolar North Atlantic. Science.

[77] Good SA, Martin MJ, Rayner NA (2013). EN4: Quality controlled ocean temperature and salinity profiles and monthly objective analyses with uncertainty estimates. J. Geophys. Res. Oceans.

