# Supplementary material for: Submesoscale modulation of deep water formation in the Labrador Sea
Source: Sci Rep. 2020 Oct 15;10:17489. doi: 10.1038/s41598-020-74345-w (PMC7566636; doi:10.1038/s41598-020-74345-w)
Supplement: Supplementary file 1 — Supplementary Information. [file 41598_2020_74345_MOESM1_ESM.docx]

**Supplementary Information for:**

**Submesoscale modulation of deep water formation in the Labrador Sea**

F. Tagklis^1*^, A. Bracco^1^, T. Ito^1^, and R. M. Castelao^2^

^1^Earth and Atmospheric Sciences, Georgia Institute of Technology, Atlanta, Georgia, USA.

^2^Department of Marine Sciences, University of Georgia, Athens, Georgia 30602, USA.

*Corresponding author: Filippos Tagklis ([ftagklis3@gatech.edu](mailto:ftagklis3@gatech.edu))

**This PDF file includes:**

**Figures S1 to S5**

**Legends for Movies S1**

**Other supplementary materials for this manuscript include the following:**

**Movies VS1**

Figure S1. 6-Year (2007-2013) mean circulation in all the runs. The color shade indicate he speed in (ms^-1^) and the vectors indicate direction of velocity, respectively. The vectors are plotted at every 0.5^o^ in longitude and latitude. (row-A) surface and (row-B) at 200 meters.


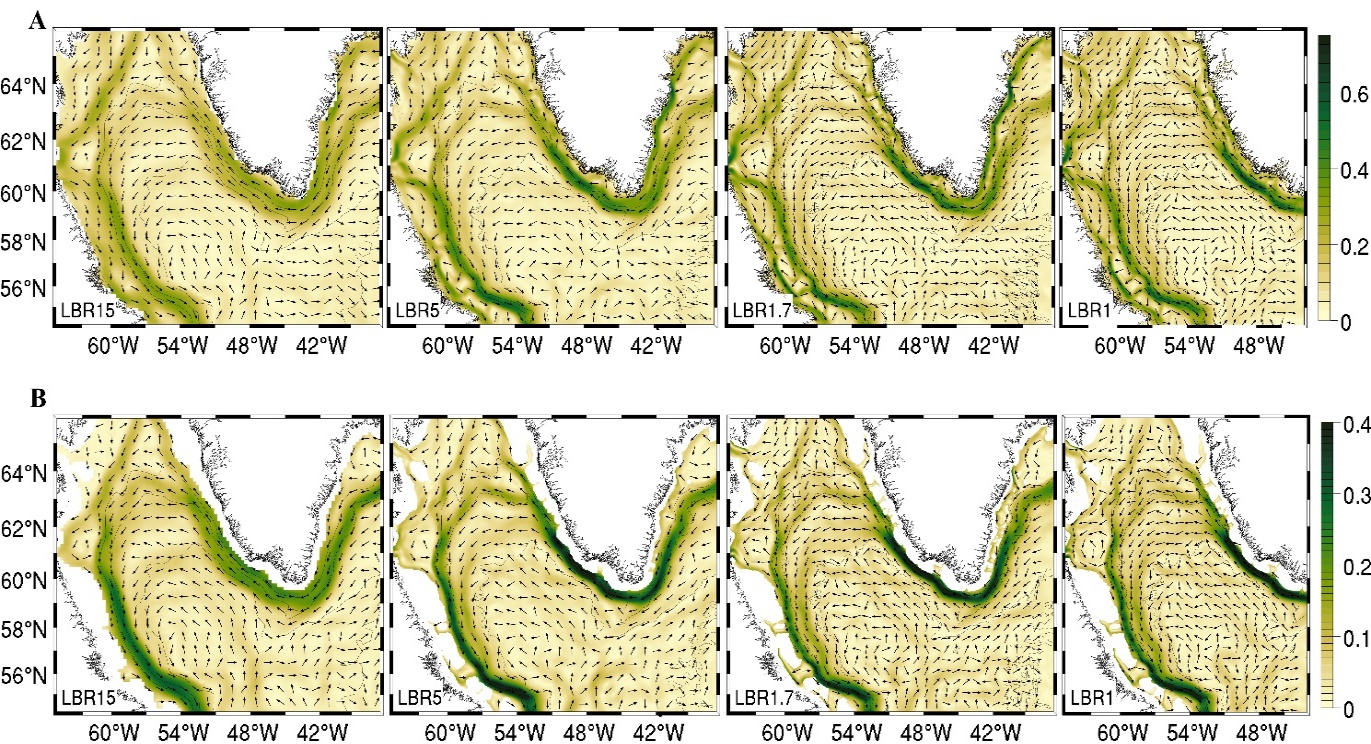


Figure S2: Snapshots of mixed-layer depth in February 2009 (times corresponding to Fig. 1) in LBR15 (A), LBR5 (B), LBR1.7 (C) and LBR1 (D). Depths greater than 1000 are color-saturated.


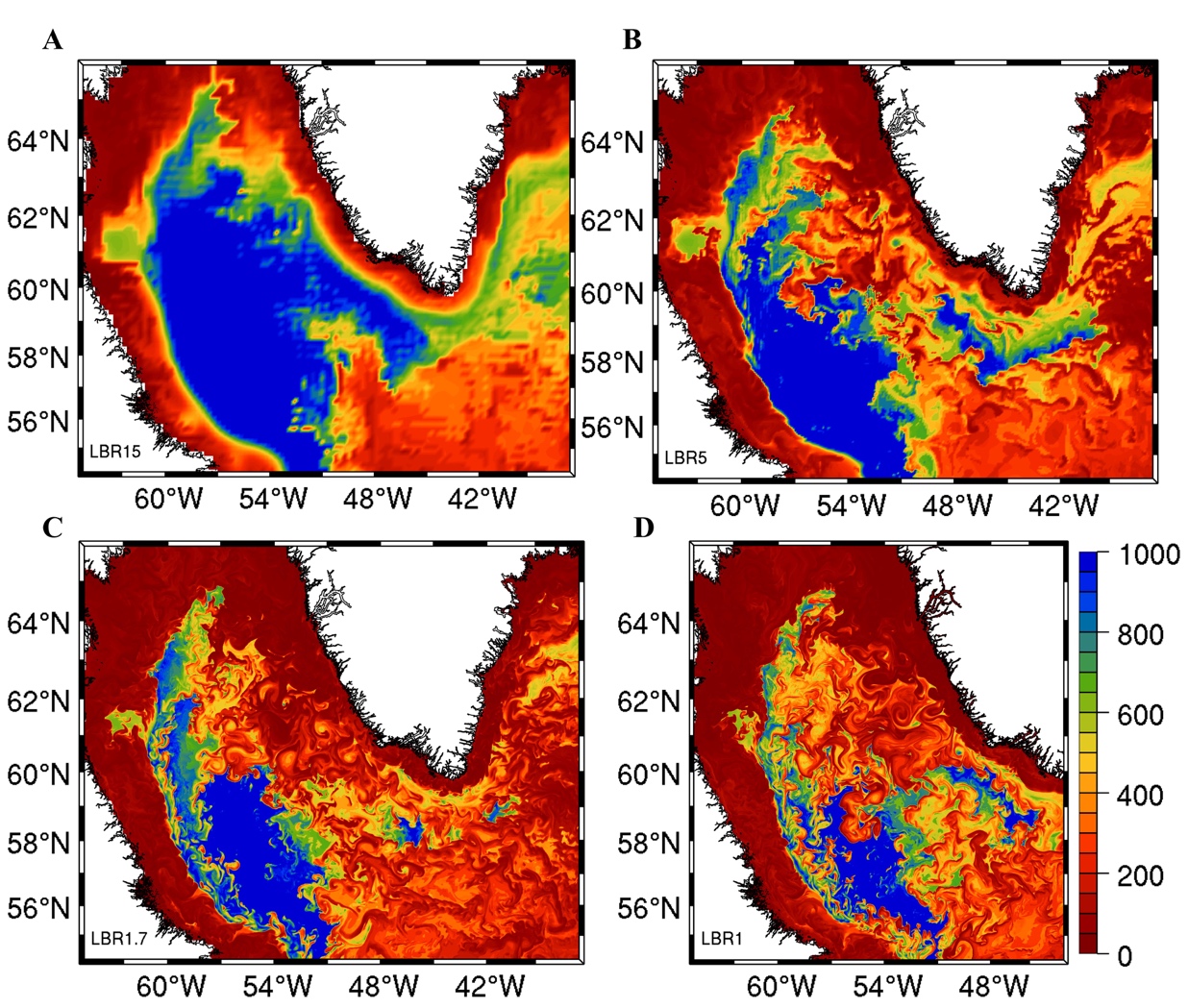


Figure S3. Cumulative surface tracer concentration in logaritmic scale for the period June 2007- May 2013 in the LBR15, LBR5 and LBR1.7 runs. Concentration values below 10^-2^ are shown in white. The same amount of tracer is introduced in all runs.


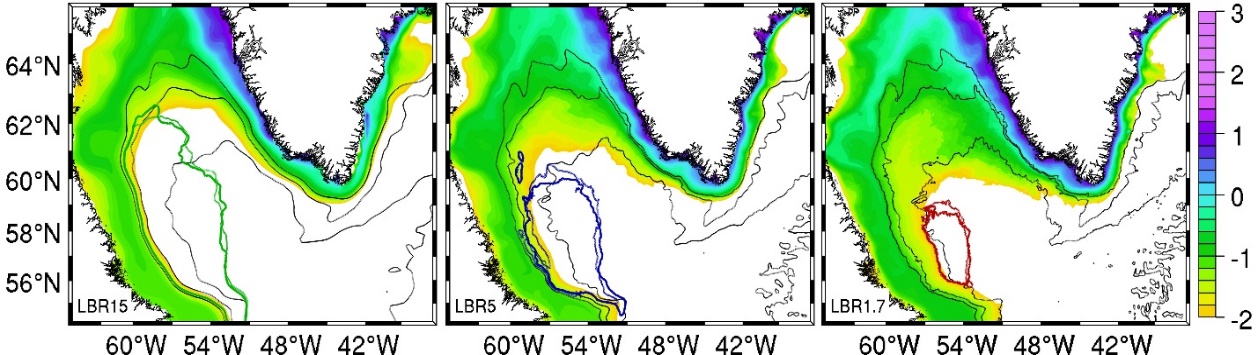

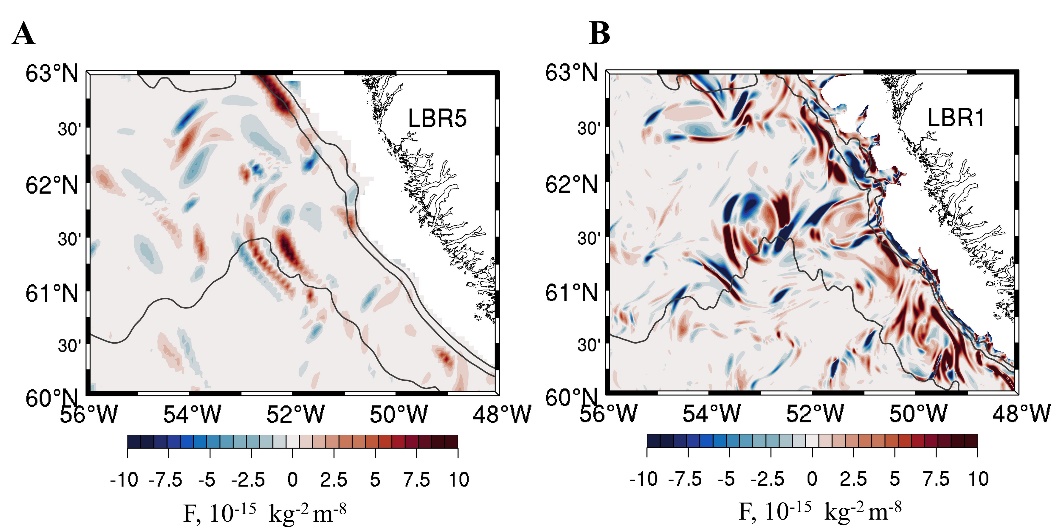


Figure S4. Frontogenetic tendency at 200 meters depth for 5km case LBR5 and 1km LBR1. Snapshots correspond to those in Figure4 (C-D).


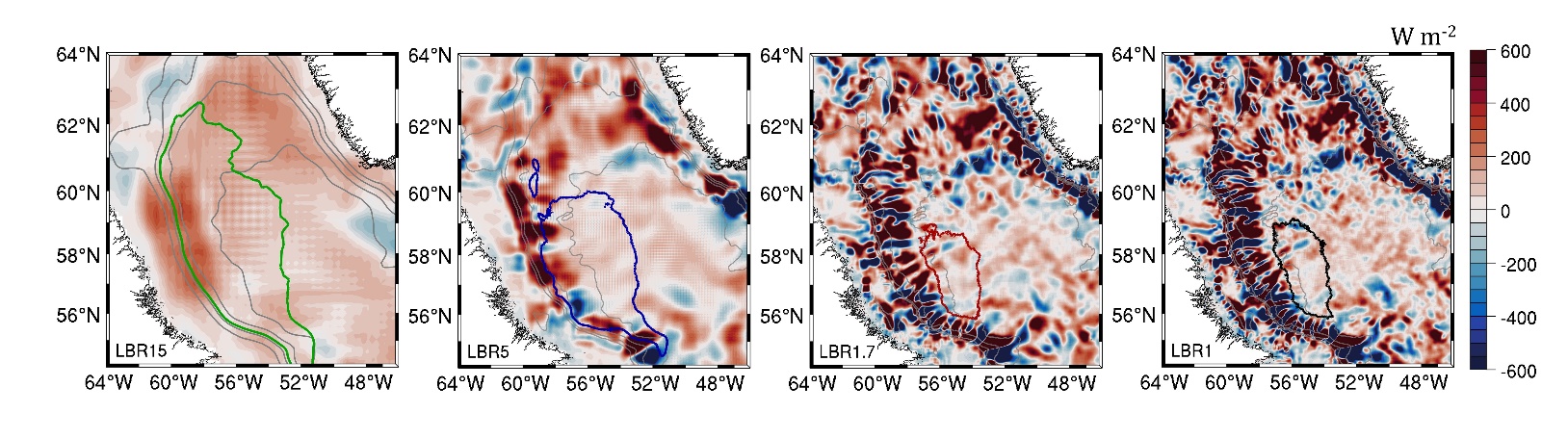


Figure S5. Depth integrated mean heat advection M (W m^-2^). Mean advective fluxes are defined as $M=-\bar{\boldsymbol{u}}\nabla\overline{C}-\overline{w}\partial_{z}\overline{C}$ where overbar represents the averaging over the period (2007-2013); ∇ is the 2 dimensional gradient operator; **u**(u,v) and w represent the horizontal and vertical components of the velocity field; $C=\rho_{o}c_{p}\theta$ represents the heat content, and ρ_ο_, c_p_ and θ are the reference density, specific heat, and potential temperature of water.

Movie VS1 (link and legend). (https://www.dropbox.com/s/6rj7lg0q0xbn0do/West_Greenland_Coast_transect.avi?dl=0)

(Top panel) Relative vorticity normalized by Coriolis (ζ/f) during February 2009, at 200 meters depth. (Bottom panel) Transect of ζ/f along the black line as it appears on top panel.
